# Anatomy of a Swedish population-scale network

**DOI:** 10.1038/s41598-025-15966-x

**Published:** 2025-08-19

**Authors:** Georgios Panayiotou, Inga K. Wohlert, Miia Bask, Mikael Bask, Matteo Magnani, Ilkka Henrik Mäkinen

**Affiliations:** 1 InfoLab, Department of Information Technology, Uppsala University, Uppsala, Sweden; 2 Department of Sociology, Uppsala University, Uppsala, Sweden; 3 Department of Economics, Uppsala University, Uppsala, Sweden

**Keywords:** Population-scale, Sweden, Social network, Multilayer network, Complex network, Complex networks, Information technology

## Abstract

With the increasing interest in large-scale social network analysis, recent research has expanded into nation-wide networks generated from administrative data. We construct a multilayer population-scale social network for Sweden using public register data from 2000 to 2017, covering approximately 8.3 million individuals aged 15 and older. The network models the social opportunity structure in Sweden across six layers: close family, extended family, household, school, neighbors, and work. We analyze the structure and connectivity patterns in the network, comparing our findings to a similar study of the Netherlands. The comparison reveals broadly similar degree distributions and small-world characteristics, but also discrepancies likely driven by differences in population density.

## Introduction

Social scientists would agree that relationships between people constitute a core matter of social research. However, for a very long time, they suffered from a lack of relational data on a scale that would correspond to the scope of their theories. As a necessity, the bulk of social research would be directed to the study of groups, that is, accumulations of people with some common source of identification, who might or might not have relationships with each other, and who can be treated as one unit.

The idea of studying human networks instead of groups was present in early sociology^1^, but systematic studies of social networks were pioneered by Jacob L. Moreno, who developed sociometry during the 1930s. In his studies, individuals’ positions within small groups were often the focus. During the second half of the 20th century, the work continued, not least along theoretical lines and with analyses of meso-level networks^2^. Still, large-scale networks could only be investigated indirectly, as in Stanley Milgram’s famous small-world experiment where the participants were asked to find their way through a nation-wide network by themselves^3^.

Still, interrelations of individuals in large-scale networks remained largely unanalyzed until network-based communication systems first appeared in the 1990s. At that point, studies using those networks also began to emerge, producing knowledge on the size of the individual networks and the intensity, and even quality, of the contacts, amongst other things. Some noted studies were made by Szell and Lambiotte^4^, whose data covered 300,000 players of a multiplayer online game, and Centola^5 ^who, alike Bond et al.^6^, experimented with the diffusion of behaviors in large-scale, online networks. While such studies witness to the possibilities of large-scale network analyses in revealing new insights into the social structures of the (virtual) world around us, however, their ability to represent offline relationships remains contested.

Another limitation affecting many network studies has been that they have most often been based on only one type of linkage (e.g., knowledge, friendship, or co-location), emanating from only one source, such as a workplace, a study class, an online network, or similar. That is not always satisfactory. If the researcher’s main interest lies in contacts between individuals, their access to each other, rather than in the qualities of a specific kind of network, various channels need to be taken into account simultaneously, such as family relations, friendships, common workplace, study class, institution, or shared neighborhood. This idea was latently present already in Granovetter’s classic study of the strength of weak ties^7^, and came to be developed during the following decades^8–12^.

Recently, researchers have started to investigate nation-wide networks built out of public registries in countries where access to good-quality population statistics is possible. Such networks can be best understood to model the social opportunity structure of that country. Using administrative data to construct a social network enables inferring formal ties representing relational states between individuals, such as kinship, coinhabitation, or collegial ties. While these relationships do not necessarily imply interaction events between individuals, they do offer interaction opportunities, through which these ties can potentially become active. As such, this affiliation-based approach allows us to empirically examine the magnitude of social opportunity structures at a national scale.

Utilizing administrative registers, van der Laan et al.^13^ developed a population-scale network describing the probable ties between individuals in the Netherlands through their family, household, neighborhood, school, and work. Building on this work, Bokányi et al.^14^ explored the structure and connectivity patterns in the Dutch network. Their investigation reveals a long-tailed degree distribution, though one contrasting with expectations for online social networks, and reconfirms the small-world nature of large-scale social networks^3^. Another recent study by Cremers et al.^15^ constituted a welcome extension by analyzing the population-wide social network in Denmark, a country that in many respects (family structure, labor market, etc.), is similar to the Netherlands. In this longitudinal study, covering the years 2008 to 2021, the authors note the varying stability between the different network layers, and the potential for earlier connections to later reemerge in other layers. A recent study by Kazmina et al.^16^ has specifically researched social segregation in the Dutch network, pointing out that segregation in social networks is much higher than that seen in residential segregation. Moreover, Hedde-von Westernhagen et al.^17^ used the Dutch nation-wide network to investigate disease spreading, while Menyhért et al.^18^ compared the connectivity and community structures between online and offline social networks of the Netherlands.

The current study aims at the construction of a nation-wide social network out of available register data in Sweden. As a first step, we focus on a network for the year 2017. Inspired by the methodology used in the study of the Netherlands^14^, we describe the anatomy of the Swedish population-scale network through three key structural measures: degree, distance, and closure. Moreover, we examine how these features vary when controlling for different sociodemographic factors, namely age, income, education level, and residence urbanization level. Finally, throughout the paper, we compare our findings with the Dutch study, investigating how the different physical geography of Sweden may affect the properties of the network. The primary focus of this work is methodological. First, we aim to assess whether a large-scale multilayer social network can be constructed from Swedish register data. Second, by comparing the Swedish and Dutch population networks’ structural properties, we examine whether networks obtained using this approach can capture meaningful features about the modeled countries. As such, we experimentally evaluate our network using the same measures to maintain consistency between the two works.

Through this study, we demonstrate that constructing a population-scale network for Sweden is indeed possible. Its accuracy, however, is constrained by the complexities imposed both by the size of the population, and by missing data, which requires us to introduce assumptions and sampling procedures when inferring potential relationships between individuals. Even so, a network constructed using this approach captures relevant features of Sweden. Comparing the anatomy of this network with the corresponding study for the Netherlands, we find notable similarities: a small-world network structure, and a degree distribution that differs from typical large-scale social networks. At the same time, the comparison highlights key discrepancies between the two countries, likely driven by population density and underlying social behavior.

## Results

We construct a multilayer population-scale opportunity network for Sweden using public registry data provided by Statistics Sweden (SCB) for the year 2017, representing residents in Sweden above the age of 15. The registry data was pseudonymized before access, and processed in line with GDPR regulations.

The network is structured into six layers, each representing a specific type of probable connection between individuals: close family (C), extended family (E), household (H), school (S), neighbors (N), and work (W); see also the “Data and methods” section for specifics on layer generation. Each layer captures a distinct aspect of social ties, with variations in the degree distributions reflecting differences in population density, societal structures, and data coverage. The close family and household layers are based on immediate and co-living relationships, while the extended family and school layers extend to broader social contexts, often influenced by generational and educational factors. The neighbors and work layers, in contrast, depend on geographic and professional proximity, respectively. Together, these layers provide a comprehensive view of the network, highlighting the diversity and complexity of social connections.

Following the approach of Bokányi et al.^14^, we describe the anatomy of the Swedish population-scale network, focusing on individuals’ degrees, distances, and closure, examining how these network properties vary between the two countries. Moreover, we investigate how these structural properties vary for different sociodemographic variables, namely age, income, education level, and urbanization level.

**Fig. 1:**
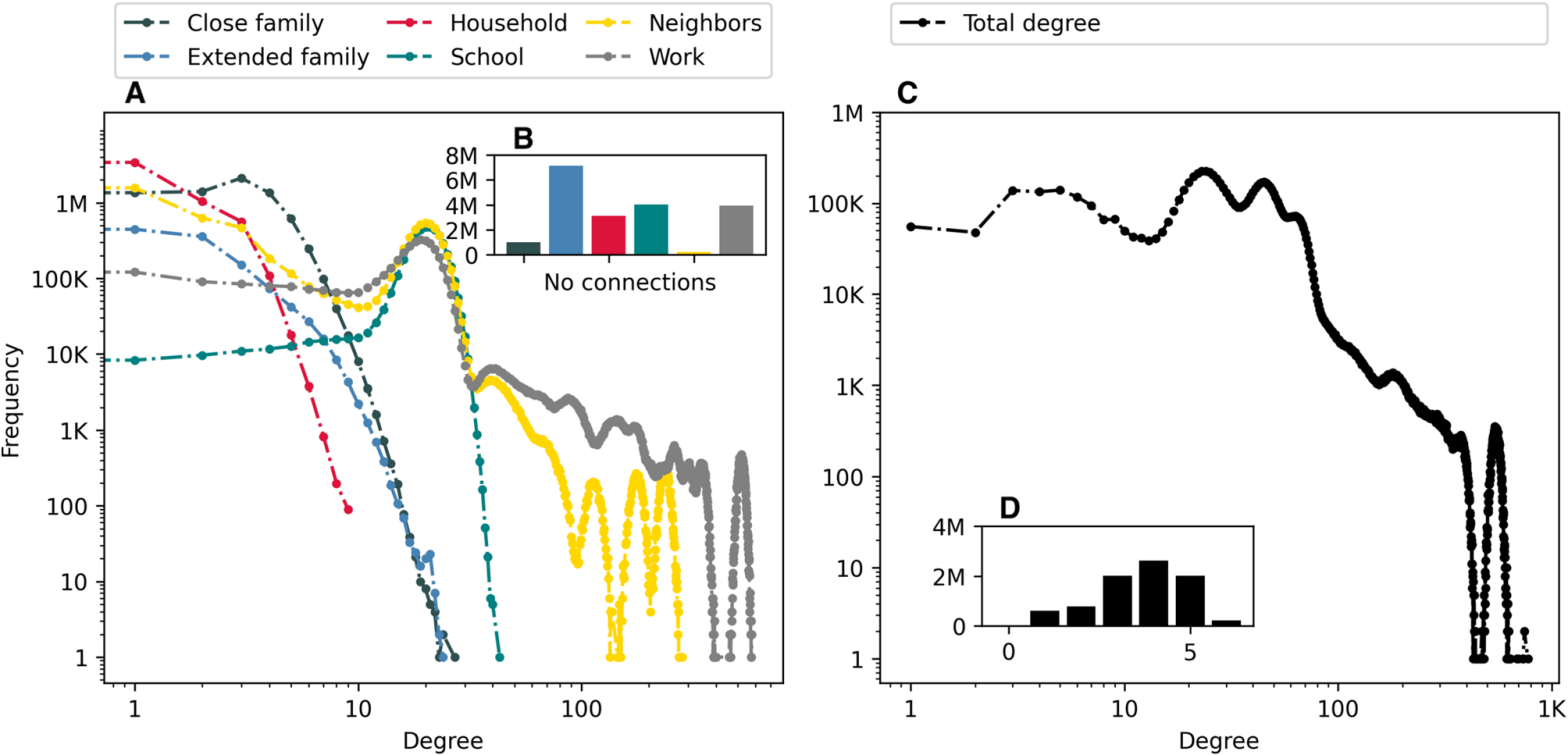
Degree distributions. (**A**) Degree distributions for individual layers (left). (**B**) Number of disconnected nodes per layer (inset left). (**C**) Total degree distribution for all layers in the network (right). (**D**) Distribution of number of layers in which a vertex has non-zero degree (inset right).

### Degree

**Fig. 2:**
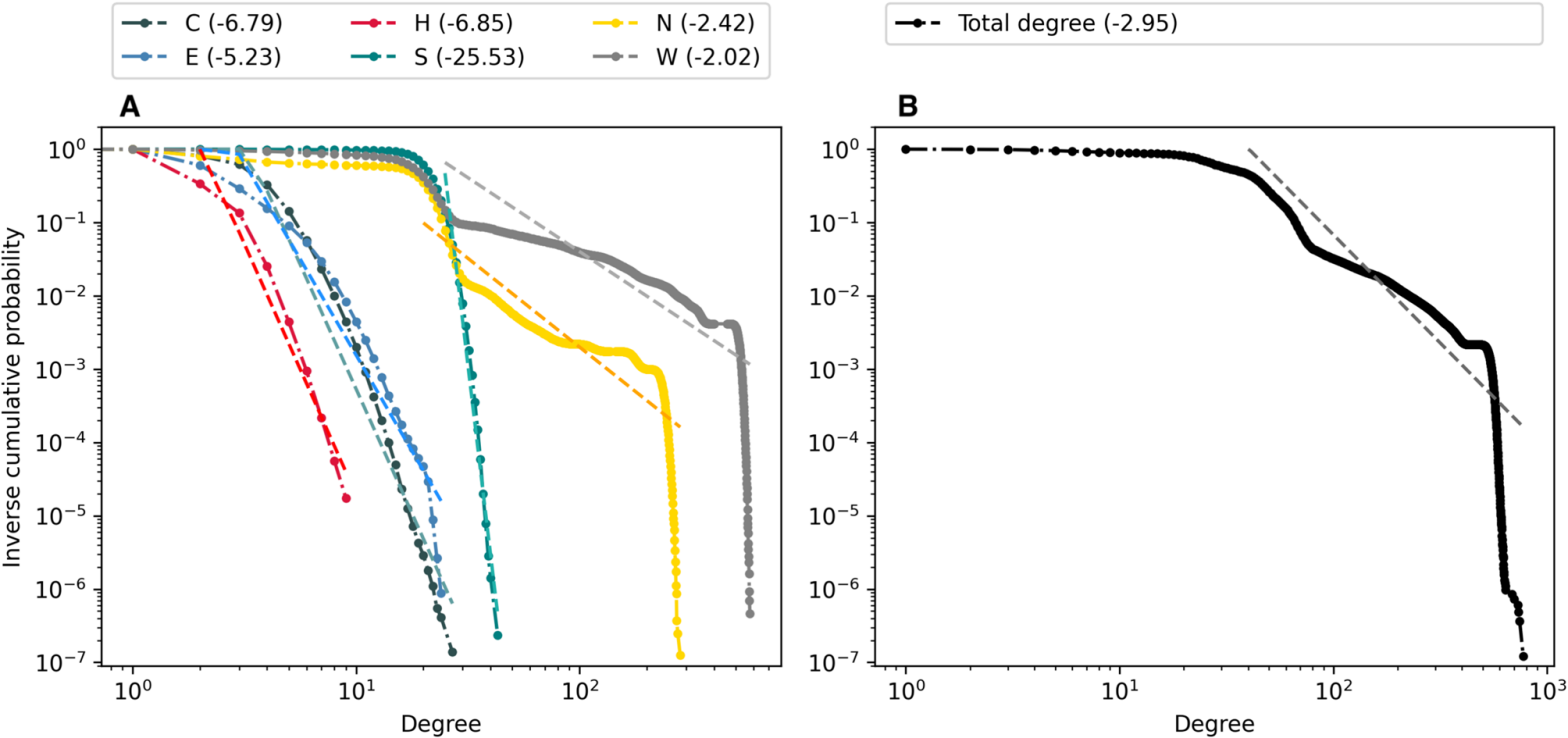
Inverse cumulative degree distributions for (**A**) individual layers, and (**B**) entire opportunity network with all layers. The values in parentheses correspond to the tail slope for each layer.

First, we examine the degree distributions in the generated population-scale network. Figure 1 illustrates the degree distributions for the six layers (1A), the number of disconnected nodes per layer (1B), the total degree distribution when all social opportunities are considered together (1C), and the distribution of the number of layers in which a vertex is connected (1D). Additionally, Figure 2 presents the inverse cumulative degree distributions, along with the tail slope for each layer and the flattened opportunity network.

Starting with the close family layer (C), the majority of individuals fall within the range of one to five degrees, with each specific degree comprising at least one million individuals. Notably, close family ties are absent for approximately one million individuals. However, cases of individuals having no connections at all are rarer in the close family layer compared to other network layers, with the exception of the neighbors layer (N). In contrast, within the Dutch network, most individuals fall within the range of three to six degrees, with each specific degree comprising at least two million individuals. Only a small number of individuals lack close family ties.

When interpreting the aforementioned findings, it is important to note that connections are established solely based on households. In Sweden, where it is common for individuals to move out of the parental home at a young age, ties are sparse across various age groups, depending on whether they can be linked to parents or children within the specified time frame. Additionally, information about family connections for immigrants is limited, except for those who arrive with their families.

In the extended family layer (E), a large proportion of individuals fall within the range of one to three degrees, with each specific degree comprising at least 100,000 individuals. However, approximately seven million individuals lack extended family ties. By contrast, the degree distribution in the Dutch network is relatively flat, ranging from one to approximately 20 degrees, with a peak at nine degrees. Furthermore, relatively few individuals lack extended family ties. It is important to note that relationships beyond the close family are significantly underrepresented in the Swedish network, as they predominantly stem from close family connections, and that generating extended family relationships requires linking at least two generations of close family ties.

In the household layer (H), a large number of individuals have either one or two degrees, with each degree encompassing at least one million individuals. Notably, approximately three million households in Sweden consist of single-person households. In the Dutch network, most individuals fall within the range of one to three degrees, with each specific degree including at least two million individuals. Compared to Sweden, the Netherlands has a lower proportion of single-person households. It is important to note that other coinhabitation arrangements, such as room sharing and student dormitories, are not included in the household layer in the Swedish network.

In the school layer (S), most individuals with connections to others have a degree of approximately 20. In the Dutch network, the degree distribution is relatively flat, spanning from one to approximately 30 degrees, with no distinct peak, unlike in Sweden. The school layer in the Swedish network connects individuals based on education level, municipality, and graduation year, with the field of study added for groups exceeding 1,000 individuals (e.g., at the university level). Notably, approximately four million individuals lack connections in the school layer.

In the neighbors layer (N), the degree distribution shows multiple peaks, reflecting differences in population density between rural and urban areas. Urban areas are characterized by higher connectivity, whereas rural areas generally have fewer connections. Notably, more than 1.5 million individuals have a degree of one, while approximately half a million individuals have a degree of either two, three, or around 20. Almost no individuals lack close neighbors. In comparison to Sweden, a smaller proportion of the population in the Netherlands resides in rural areas, a distinction that is reflected in the degree distributions when comparing the two countries.

In the work layer (W), most individuals with connections to others have a degree of approximately 20. However, around four million individuals lack connections in the work layer. The peak at approximately 500 degrees corresponds to large single-location workplaces, such as factories. In the Dutch network, the degree distribution is relatively flat, ranging from one to approximately 100 degrees.

Regarding the degree distribution of the flattened opportunity network, we observe that most individuals have a degree around 25. Contrary to expectations for large social networks^19,20^, we note that the degree distribution of the Swedish network does not resemble the well-studied power-law or log-normal distributions. In Figure 2 we note that the tail slope for the total degree distribution, as well as for most individual layers, is well above 2. Similar effects can be seen in the Dutch population network, although by contrast, most individuals have a degree of around 40. This discrepancy can be attributed to the great difference in population density between Sweden and the Netherlands.

However, it is critical to note how the total degree distribution is also affected by the edge sampling procedures behind the affiliation-based layers. Within these layers, contrary to a social network of observed interactions, an individual receives a degree that partially reflects the size of their class, neighborhood, or workplace. This effect is particularly visible for the neighbor and work layers, where we can note a few low-frequency, high-degree peaks which correspond to individuals in large residential and workplace groups, respectively.

**Table 1:** Layer comparison. (A) Pearson correlation between node degrees in different layers, $p < .0001$ (left). (B) Edge overlap ratio between layers, normalized per row over diagonal and per column under diagonal (right).

Layer node degree correlation						
	**C**	**E**	**H**	**S**	**N**	**W**
**C**	1.00	0.15	0.33	0.25	−0.12	0.10
**E**		1.00	0.13	0.02	−0.04	−0.04
**H**			1.00	0.06	−0.16	0.01
**S**				1.00	−0.07	0.11
**N**					1.00	−0.01
**W**						1.00

Layer edge overlap ratio						
	**C**	**E**	**H**	**S**	**N**	**W**
**C**	1.00	0.01	0.34	0.00	0.12	0.01
**E**	0.10	1.00	0.02	0.00	0.01	0.00
**H**	1.00	0.01	1.00	0.00	0.35	0.01
**S**	0.00	0.00	0.00	1.00	0.00	0.00
**N**	0.05	0.00	0.05	0.00	1.00	0.00
**W**	0.00	0.00	0.00	0.00	0.00	1.00

Finally, according to Table 1, the correlation and edge overlap ratio between the close family layer (C) and the household layer (H) are higher than those of all other layer pairs. This finding is not surprising, as the household layer captures relationships between co-resident family members. Moreover, most of the other edge overlap ratios are small, indicating that each layer provides unique information about individuals’ social opportunity ties that is not present in the others. This is not surprising either. Furthermore, the correlations and edge overlap ratios between the layers for Sweden and the Netherlands appear to be of similar magnitudes.

### Components and shortest path length

**Table 2:** Network characteristics for various layer combinations. The number of vertices and edges is reported in millions, and the number of components in thousands. GC is the relative size of the giant component related to the network size, D is the diameter of the giant component and $\overline{d}$ the average shortest path of the giant component.

Layers	Vertices (M)	Edges (M)	Components (k)	GC	D	$\overline{d}$
C	7.25	21.76	1154.77	0.13	337	119.89
C+E+H	7.32	24.49	1152.42	0.15	330	106.45
C+E+H+N	8.22	128.61	172.68	0.94	34	8.78
C+E+H+N+S	8.25	214.67	59.25	0.98	17	5.84
C+E+H+N+S+W	8.26	329.16	45.06	0.99	15	5.11

We continue by examining how the various layers contribute to the connectivity patterns among Swedish residents in the network, focusing on their roles in forming a giant component and shortest paths. As Bokányi et al. point out, the affiliation-based edge generation mechanism can produce highly clustered layers^14^. Therefore, it is worth assessing whether the population network has small-world properties, and to what extent these effects are generated by distance-bridging edges, also referred to as network wormholes^21^.

Table 2 displays network characteristics for various layer combinations, highlighting several noteworthy aspects. First, as expected, the average shortest path length decreases as the number of layers increases. However, on the other hand, this decrease is less pronounced when the extended family (E) and household (H) layers are added to the close family (C) layer. Starting with the close family layer, two randomly selected individuals are approximately 120 steps apart. This number decreases only slightly to about 106 when the extended family and household layers are included. This finding contrasts sharply with results from the Netherlands, where the average shortest path length decreases significantly when incorporating the close family, extended family, and household layers.

One reason for our finding is that, in contrast to the Netherlands’ study, approximately seven million individuals in the population network of Sweden lack extended family ties. Additionally, other co-living arrangements, such as roommates and student dormitories, are not included in the household layer of the Swedish network. However, when the neighbors (N), school (S), and work (W) layers are added to the close family (C), extended family (E), and household (H) layers, the average shortest path length decreases to 5.11 (cf., Frigyes Karinthy’s concept of six-degrees-of-separation), which is only slightly higher than the corresponding figure for the Dutch network (4.64).

Second, which is a direct reflection of the previous finding, is that the relative size of the giant component, compared to the size of the network, increases alongside the number of layers. When the extended family (E) and household (H) layers are added to the close family layer (C), only 15% of the vertices form a giant component; instead, there are a lot of smaller components. However, when adding the neighbors layer (N) to the network, the relative size of the giant component increases to 94%. The main reason for the large increase of the giant component’s relative size is that relationships beyond the close family are considerably underrepresented in the Swedish network, largely an effect of the aforementioned limitation of generating extended family relationships by linking at least two generations of close family ties. This contrasts with findings from the Dutch network, where the extended family layer plays a primary role in connecting an overwhelming majority of individuals.

**Fig. 3:**
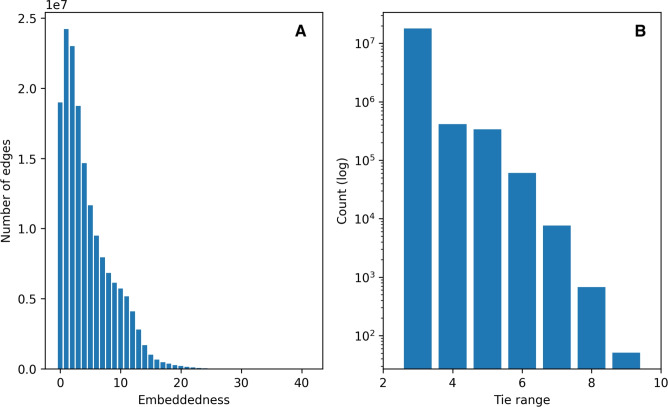
Embeddedness and tie range. (**A**) Embeddedness distribution for all edges in the network (left). (**B**) Tie range for all edges with embeddedness zero, where both endpoints’ degree $>1$ (right).

To understand whether the observed small-world effects in the Swedish network is produced by edges acting as local bridges that shorten the distances between individuals, we study the edges’ embeddedness (i.e., the number of common neighbors between an edge’s endpoints^22^), and tie range (i.e., the second-shortest path^21^) between endpoints of those edges where embeddedness is zero.

In Figure 3A, we observe an overall low edge participation in triangles throughout the network. Approximately one third of the edges in the network receives embeddedness values less than five, suggesting loosely connected local clusters. Interestingly, we find that close to 19 million edges ($\approx$ 5.7% of all edges) receive an embeddedness score of zero. These edges represent local bridges that shorten distances within the network, and as a result can produce small-world-like network structures.

Measuring the tie range for these edges (Figure 3B), we find that the overwhelming majority has an alternate path distance under six, suggesting that these ties do not act as major structural bridges. Just over 69,000 of these edges ($\approx$ 0.02%) represent so-called network wormholes, that is, long-range network ties with a distance of at least six, with a small number of edges having a tie range up to nine. While in our population network the wormhole ratio is overall low, and significantly lower than the Singapore Twitter network presented in the seminal tie range study (0.46%)^21^, this proportion is larger than in the Netherlands’ population network, where only 0.02% of the total edges represent local bridges, and the percentage of network wormholes shrinks down to 0.0011%^14^.

### Clustering and excess closure

**Fig. 4:**
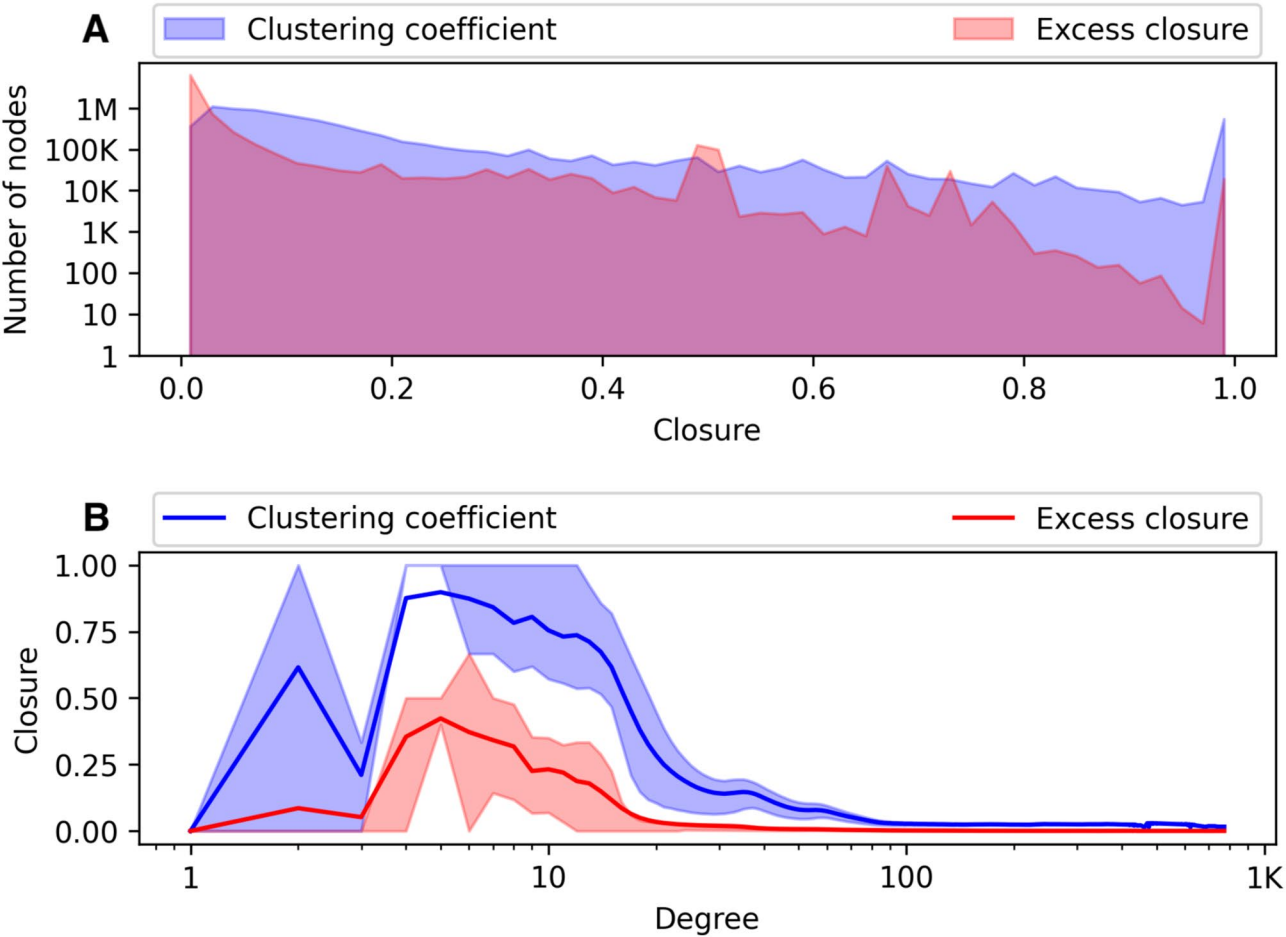
Local clustering coefficient and excess closure. (**A**) Distributions of clustering coefficient and excess closure values (above). (**B**) Average clustering coefficient and excess closure per vertex degree. Shaded areas correspond to the 25th and 75th percentile (below).

Next, we consider closure, an important structural measure of the network affecting individuals’ access to social opportunities and information^23,24^. As the affiliation-based nature of the layers can produce highly clustered structures, we calculate two measures. First, we measure the clustering coefficient over the flattened opportunity network, to identify the closure density around individuals. Second, we consider excess closure^14^, which captures to what extent the observed closure arises from the integration of multiple social contexts, by distinguishing between single-layer and multi-layer triangles formed around a vertex.

In Figure 4A, we notice a relatively strong overlap between the distributions for the local clustering coefficient and the excess closure in the network, particularly for values of closure less than 0.5. A large amount of the individuals in the network receive closure values of zero, which is a direct result of the aforementioned overall low triangle participation (cf., Figure 3A). However, we also report a large amount of vertices with clustering coefficient values of one. Similarly to the Dutch study, this effect can be attributed to affiliation-based nature of the school, neighbor, and work layers, as they can generate larger degrees compared to the familial layers.

Plotting the average clustering coefficient and excess closure scores over the vertices’ degrees in Figure 4B, we observe a peak in the closure values for individuals with degree five, before both start dropping significantly at around the 15-degree mark. Interestingly, for vertices having a degree between four and five, the overwhelming majority receives a clustering coefficient value of one, while the rest receive values closer to zero; note that the average value is lower than the respective 25th percentile. For individuals with a higher degree, we note both the low amount of triangles formed with respect to their number of connections in the network, and the tendency for those triangles to be contained within a single layer, given that the average values for both clustering coefficient and excess closure approach zero as the degree increases.

In our network, we observe that close to 100,000 vertices receive an excess closure value of one. Considering also the higher average excess closure values for individuals with a smaller amount of connections, this indicates the need to leverage multiple social contexts when forming triadic structures and, in turn, communities. However, the average excess closure values remain overall low in the network when controlling for degree, suggesting a strong tendency to form highly clustered structures within a single layer, though this can be attributed to the large degrees generated by the affiliation-based layers. This is in contrast to the Dutch network, where all individuals receive excess closure values less than 0.9, although the respective average excess closure values also remain low when controlling for degree.

### Network positions at various stages of life

**Fig. 5:**
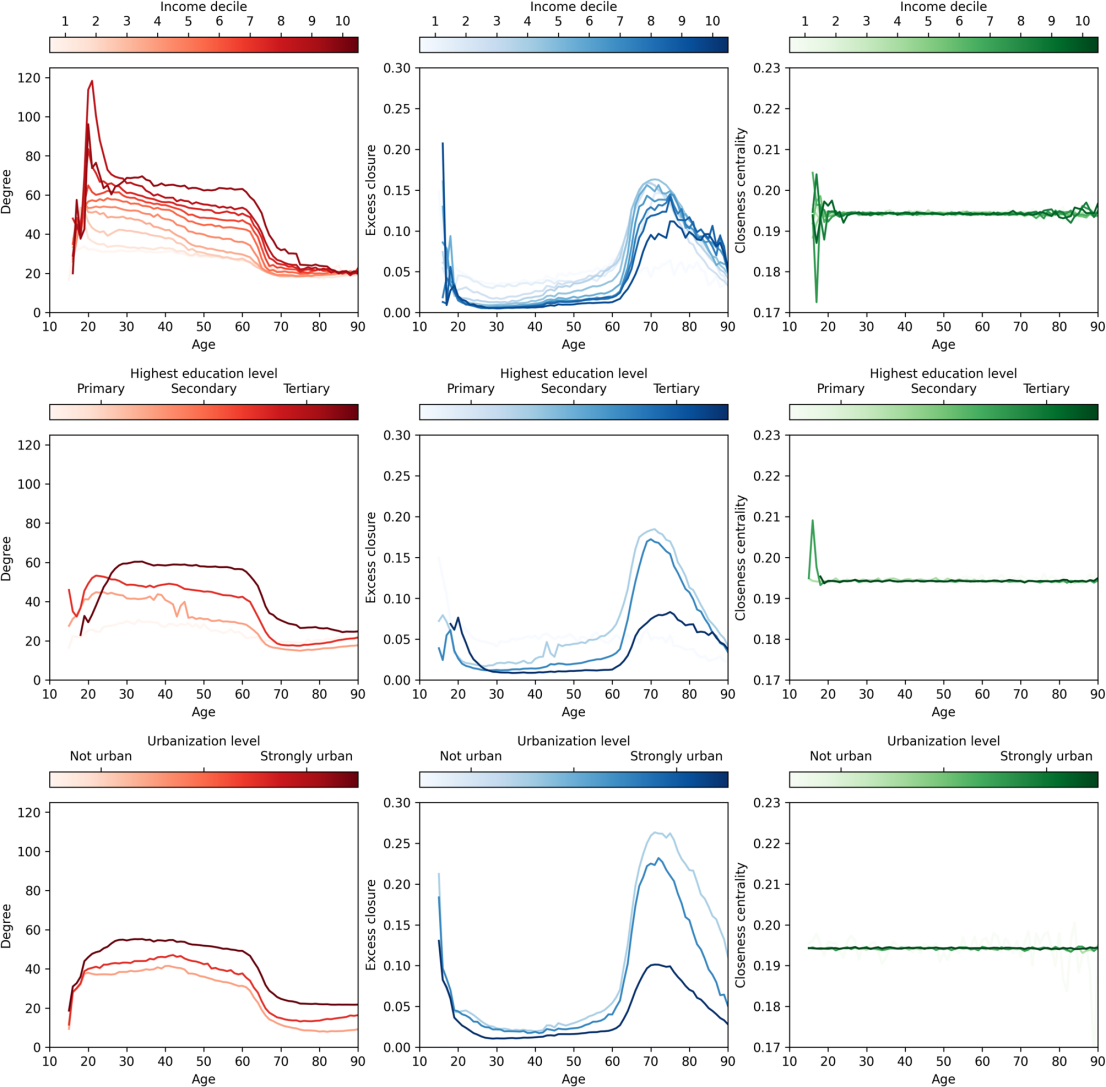
Degree, excess closure, and closeness centrality in different stages of life for various demographic groups. Average degree (red), average excess closure (blue) and closeness centrality (green) over various levels of income (top row), education (middle row) and urbanization (bottom row).

Finally, we investigate the change in individuals’ positions in the network as a function of various sociodemographic factors: their age, level of income, education, and residence urbanization. As the constructed nation-wide network represents a specific snapshot in time, namely, individuals’ family, work, education, and residence status in 2017, we do not aim to analyze changes in individuals’ social positions over the years. Instead, we aim to identify differences across various subpopulations, using the available demographic information in the population registers. In Figure 5, we plot the average degree (red, left column), excess closure (blue, middle column), and closeness centrality (green, right column) per age. We additionally identify the differences between individuals with respect to their level of income (top row), highest level of education (middle row), and residence location (bottom row).

We observe an interesting effect from the degree plots: generally, higher levels of income, education, and urbanization lead to a higher average degree, and thus, more opportunities to connect with individuals. We observe a peak in the average degree lying just over age 20, as around that age, people are more likely to form a large amount of connections through institutions for higher education and work. This finding is in line with the trends seen in the Dutch network, where the peak in average degree is also seen around age 20.

Moreover, values for closeness centrality are extremely similar for our network, regardless of age, income, education, or location of residence. The only exceptions can be seen in the relatively strong fluctuations for individuals under 18 and over 80 years old, a result of the limited data available for those ages. This is a notable discrepancy with the Netherlands’ network, where similar tendencies to the respective degree plots can be observed; namely, a peak around age 20, and a gradual decrease of the average closeness centrality with age.

We also observe the higher values of excess closure noted in the Dutch study, for both individuals over age 60, and teenagers and young adults below age 20. This finding is expected, considering that teenagers below age 18 are mostly connected through the family, school, and neighbor layers. The excess closure reaches a local minimum around ages 25-30 when more social opportunities are added, and, similarly to the Netherlands’ network, gradually increasing before reaching a final peak in later ages.

However, we note larger differences in excess closure between different levels of urbanization. This finding can be explained by the construction process behind the neighbors layer: compared to urban area residents often living in multi-apartment complexes (and being potentially connected to more neighbors), the network captures less information about the neighborhoods of rural area residents. Similar variations are also visible for various levels of education, albeit smaller in difference.

## Discussion

In this study, we demonstrate that it is possible to construct a population-scale network for residents of Sweden using public register data, representing Sweden’s social opportunity structure. We also show that a network constructed using this approach can capture relevant features of a country, by comparing the Swedish network’s anatomy with the respective study of the Netherlands^14^. The design of this study, including the similar setting for layer construction, allows direct comparison of the observed features of both networks.

Analyzing the network structure confirms several interesting trends also noted in the Dutch study. First, we observe an unusual degree distribution not matching expectations for large-scale social networks, with most vertices receiving a degree around 20. Second, we note the small-world characteristics of the network. Finally, when controlling for age, we note that higher income levels typically coincide with a higher amount of connections.

However, the Swedish network also has differences to the Dutch one, particularly with regards to the embeddedness and closure distributions. While these discrepancies are partially due to the population density difference between the two countries, the precision of some of our results is also likely to be affected by missing data, as well as the design and sampling choices behind the creation of the layers. For example, we can only infer relatively few extended family relationships compared to the rest of the layers, using the limited time range of available registry data (2000-2017). This has implications on the network structure when we consider only a subset of the layers; for example, in the Netherlands’ network, extended family ties are able to connect most of the residents, whereas in our case, the neighbors layer has a critical role in forming the giant component.

Furthermore, using administrative data to infer relationships comes with its own limitations, as Bokányi et al. also point out^14^. While using registry data allows us to capture formal ties between Swedish residents, we are not able to assess how strong these ties are, especially for affiliation-based ties, nor infer relationships for significant informal ties, for example, friendships outside school and work, or mutual participation in clubs and student organizations.

We also note the computational limitations in processing networks of such vast scale, which have also been highlighted by previous studies on population-scale networks^13,15^. Computing simple network measures (e.g., degree and embeddedness) for flattened representations of a multilayer network is possible in some network analysis software, even for networks representing the entire population of a country. However, more computationally expensive tasks, particularly those requiring the calculation of paths (e.g., closeness centrality and tie range), require approximate methods due to the enormous amount of vertices and edges to be processed. Analyses requiring still more complex tasks such as community detection, or methods specific to multilayer networks, may require a task-specific software solution, as multilayer network analysis software often face issues processing networks of such scale^25^.

Nevertheless, this work provides tremendous potential opportunities to study large-scale societal effects in Sweden. Population-scale networks have already been used for studies on segregation^16^ and epidemics^17^, while they also provide a realistic large-scale social network for other societal studies, for example, on polarization, social influence and opinion formation, mobility, and public health. In addition, attributed networks of this scale can be used as benchmark datasets for evaluating computationally expensive complex network analysis methods, such as community detection with fairness constraints^26^.

Future work on the Swedish population network can also consider the geographic properties of the network to improve the process behind inferring relationships, perhaps by employing a physical distance-based function to model the existence probability of a tie^27^. This approach can be particularly useful for the neighbor, school, and work layers, where the physical distance between individuals can affect the strength of a tie, or its likelihood of activation. Finally, an important direction for future research, albeit one falling outside the scope of this study, is conducting a longitudinal analysis leveraging the entire range of available register data for Sweden. This includes studying the layers’ temporal evolution, similarly to the analysis for the Danish nation-wide network^15^, and the stability of community structures over time.

## Data and methods

In this section, we explain in more detail the design behind each layer of the network, along with the measures used for its characterization throughout the Results section.

### Vertices

For the construction of the network, we use pseudonymized registry data from the Longitudinal Integration Database for Health Insurance and Labor Market Studies (LISA), maintained by Statistics Sweden (SCB), on Swedish residents above the age of 15 between the years 2000-2017. While we use the entire range of years to derive family relationships, the vertices of the network correspond to over 8 million individuals for which there is registry information available in the year 2017.

### Layers

In order to follow the construction of the Netherlands’ network^14^, we construct six layers: close family (C), extended family (E), household (H), school (S), neighbors (N), and work (W). We infer inter-layer relationships based on pseudonymized personal identifiers for each vertex. The processes behind the construction procedure of each layer are summarized below^28^.

**Close family** For the close family layer, we infer parent/child, partner, and sibling relationships, using the age difference between individuals assigned to the same family in the dataset.

As we only have information for individuals 15 years old or older, using data only for 2017 would result in a very loosely connected network. Therefore, data from all available years (2000-2017) were aggregated in order to construct the family layers. However, we solely consider the relationships between individuals for which there is registry information available in 2017, as the registry data change over the years due to deaths, immigration, and individuals reaching the age of 15.

Information about family connections is limited for immigrants who do not arrive together with their family. Furthermore, as connections can only be made based on households, and it is common in Sweden to move out of the parental house at a young age, connections are sparse for certain age groups depending on the ability to connect them with parents or children in the given time frame.

**Extended family** To construct the extended family layer, we construct grandparent/grandchildren, uncle/aunt, niece/nephew, and cousin relationships. All of these connections are based on the close family connection, so for example, a grandparent is the parent of a parent. Since we are able to infer extended family edges by connecting at least two generations of close family ties, the relations outside the close family are highly underrepresented in the overall network.

**Household** The dataset also contains identifiers of individuals residing in the same household, provided that they are family relatives. Using this information, we construct edges for the household layer. Note that other coinhabitation, for instance, sharing an apartment with a roommate that is not a family relative, is not captured in this layer.

**School** The school layer includes edges between individuals with matching education level, municipality, and graduation year. If such a group of people contains more than 1,000 individuals (e.g., at university level), we also consider their line of education. The graduation year data corresponds to the latest passed course; as a result, we are also able to generate edges between students currently attending the same programme, as they will have the same value for their graduation year. We do not consider groupings larger than 10,000 individuals, as for these cases we do not have enough information to reliably infer opportunity edges. This decision mostly affects high school graduates from municipalities with many graduates per year (e.g., central Stockholm).

Since the amount of people belonging to the same school grouping can be very large, we sample the number of connections needed to portray the underlying distribution of individuals in that group. This is done by calculating the amount of connections each individual is expected to have by fitting to a power-law for smaller groups, and a linear function for larger groups^28^.

**Neighbors** This layer models neighbor information for people living on the same property, based on a unique property identifier (Swedish: Fastighetsbeteckning) in the dataset. Since a single property in the data can include information about hundreds of residents in larger properties (e.g., multiple apartment buildings and student corridors), we can infer edges about neighbors in the same building. As the number of people living in the same property can also be large, especially for urban areas, we infer edges for the neighbors layer by sampling to fit the vertices’ distribution in that property, using the same principle as for the school edges.

**Work** The work layer includes information of individuals working for the same company (or at the same workplace within a company, in case of larger companies with multiple locations). For this, the main source of income for each individual in the network is utilized. For small companies (less than 200 employees), connections are based on the same sampling procedure as for the neighbors and school layers. For larger companies the grouping is further subdivided into workplaces (locations of a company).

### Network model and measures

Here, we briefly introduce the network measures used throughout the Results section. To compare our findings to the study of the Netherlands^14^, we consider equivalent definitions of the network model and descriptive measures. Unless otherwise stated, we use the NetworKit library^29^ to process our population network.

**Multilayer networks **Following the definition of Kivelä et al.^10^, we represent the adult population of Sweden in 2017 as an undirected, single-aspect multilayer network $M=(V_M,E_M,V,{\textbf {L}})$, where the vertex set *V* contains all individuals in the network ($\approx ~8.3$ million vertices). There are six layers in the layer set ${\textbf {L}}=\{C,E,H,S,N,W\}$, corresponding to the types of social opportunities described above. The node set $V_M\subseteq {V \times {\textbf {L}}}$ contains the individuals participating in each layer. Although the original definition allows for inter-layer edges, in our case the edge set $E_M$ only contains undirected intra-layer relationships between individuals connected in each social context (and thus layer): $E_M \subseteq \{(u,l,v,l): (u,l),(v,l) \in V_M, u \ne v, l \in {\textbf {L}}\}$. We do not consider coupling edges (i.e., edges connecting the same vertex in different layers) when calculating the network measures.

For the definition of the following network measures, *M* is represented as an undirected supra-adjacency matrix *A*, as follows (note that $A_{uvl}=A_{vul}$, as *M* is undirected):

1 $$\begin{aligned} A_{uvl}= {\left\{ \begin{array}{ll} 1 & \text {if } (u,l,v,l) \in E_M,\\ 0 & \text {otherwise}\\ \end{array}\right. } \end{aligned}$$

**Degree** The degree of a vertex $u \in V$ in a specific layer $l \in {\textbf {L}}$ is given by:

2 $$\begin{aligned} k_{u,l}=\sum _{v \in V} A_{uvl}. \end{aligned}$$

We also calculate the total degree of *u* over all layers similarly to Bokányi et al.^14^:

3 $$\begin{aligned} k_{u}=\sum _{l \in {\textbf {L}}}\sum _{v \in V} A_{uvl}, \end{aligned}$$

and the number of unique neighbors of *u*, corresponding to the vertex degree for the flattened multilayer network, that is, aggregating all the layers into a monoplex network:

4 $$\begin{aligned} k_{u}'=\left| \left\{ v \in V:\sum _{l \in {\textbf {L}}} A_{uvl}>0\right\} \right| . \end{aligned}$$

**Paths and components** Connected components in an undirected network are subgraphs, such that any pair of vertices $u,v \in V$ in the component is connected by a path. We consider a path to exist between two vertices if the corresponding nodes in $V_M$ are connected on any of the layers, that is, $\exists l_1, l_2 \in {\textbf {L}}$ such that $(u,l_1,v,l_2) \in E_M$. We refer to the largest connected component in the network as the giant component.

Similarly to Bokányi et al.^14^, we use the teexgraph library^30^ to calculate all values in Table 2; namely, the number of vertices, edges and components in the network, the relative size of the giant component, the exact diameter of the giant component (i.e., the shortest path length between the two most distant vertices in the component), and an approximation of the average shortest path length between all vertices in the giant component.

**Embeddedness and tie range** In a monoplex network, the embeddedness for an edge is given as the number of common neighbors between the edge’s two endpoints^22^. Practically, this can also be calculated as the number of triangles that an edge participates in. For a multilayer network, we also consider triangles spanning multiple layers. In this case, the embeddedness for an edge between vertices $u,v \in V$ in any layer is given by:

5 $$\begin{aligned} embeddedness_{u,v}=\sum _{l_1,l_2 \in {\textbf {L}}}\sum _{w \in V} A_{uwl_1} \cdot A_{vwl_2}. \end{aligned}$$

For all edges not participating in triangles, we also calculate the tie range, which is the the second shortest path between *u* and *v*^21^. To achieve this, we obtain the shortest path using bidirectional Breadth-First Search (BFS), after excluding edges between the two vertices.

**Closeness centrality** Closeness centrality for a vertex *u* is the reciprocal of its average farness to all vertices:

6 $$\begin{aligned} closeness_u=\frac{|V|-1}{\sum _{v \in V, u \ne v} d(u,v)}, \end{aligned}$$

where *d*(*u*, *v*) is the shortest path length between those vertices; as above, we consider paths spanning multiple layers.

Calculating exact closeness values requires computing shortest paths between all vertices, which is computationally expensive for a large network. Therefore, we use the teexgraph library^30^ to measure an approximation of closeness centrality for all vertices, for a sample of 0.03% of the giant component.

**Local clustering coefficient and excess closure** The local clustering coefficient for a vertex *u* is defined as the ratio of the number of triangles spanning over all layers that *u* participates in, over the number of neighbor pairs for *u*:

7 $$\begin{aligned} lcc_u=\frac{|\{(u,w,v):(u,w), (u,v), (w,v) \in E'_M \} |} {k'_u\cdot (k'_u-1)}, \end{aligned}$$

where $E'_M$ is the edge set corresponding to an aggregated version of the network, for example, $E'_M=\{(u,v): (u,l,v,l) \in E_M, l \in {\textbf {L}} \}$, and $k'_u$ is the number of unique neighbors of *u*.

Excess closure is introduced by Bokányi et al.^14^ as a closure metric able to distinguish between different types of multilayer triangles; namely, pure triangles where the edges all belong to a single layer ($T\_pure_u=\sum _{l \in {\textbf {L}}} T_u^{lll}$) and triangles spanning multiple layers ($T\_unique_u=\sum _{l_1,l_2,l_3 \in {\textbf {L}}} T_u^{l_1l_2l_3}$), where $T_u^{lll}$ and $T_u^{l_1l_2l_3}$ are the counts of pure and multilayer triangles around vertex *u*, respectively.

Its calculation also requires the number of alter tie pairs for *u*:

$$\begin{aligned} P_u= {k_u \atopwithdelims ()2} - \sum _{v \in neighbors(u)} {\sum _{l \in {\textbf {L}}} A_{uvl} \atopwithdelims ()2}, \end{aligned}$$

where $neighbors_u=\{v \in V: (u,l,v,l) \in E_M, l \in {\textbf {L}}\}$ is the set of vertices neighboring *u* in any layer.

Finally, the excess closure can be calculated as:

8 $$\begin{aligned} c\_excess_u=\frac{T\_unique_u - T\_pure_u}{P_u-T\_pure_u}. \end{aligned}$$

## Data Availability

The data supporting the findings of this study are available from Statistics Sweden (SCB) for qualified researchers, but they are not publicly available for legal reasons. For more information on data access, we refer to the SCB micro-data website (https://www.scb.se/en/services/ordering-data-and-statistics/microdata/). Other questions related to data access can be addressed to the corresponding author, or the micro-data hub of SCB (mikrodata@scb.se).

## References

[1] Simmel, G. *Soziologie* (Duncker & Humblot, 1908).

[2] Hedström, P., Sandell, R. & Stern, C. Mesolevel Networks and the Diffusion of Social Movements: The Case of the Swedish Social Democratic Party. *American Journal of Sociology* **106**, 145–172, 10.1086/303109 (2000). Publisher: The University of Chicago Press.

[3] Milgram, S. The small world problem. *Psychology today* **2**, 60–67 (1967).

[4] Szell, M., Lambiotte, R. & Thurner, S. Multirelational organization of large-scale social networks in an online world. *Proceedings of the National Academy of Sciences* **107**, 13636–13641, 10.1073/pnas.1004008107 (2010). Publisher: Proceedings of the National Academy of Sciences. 10.1073/pnas.1004008107 PMC2922277 20643965

[5] Centola, D. The Spread of Behavior in an Online Social Network Experiment. *Science* **329**, 1194–1197, 10.1126/science.1185231 (2010). Publisher: American Association for the Advancement of Science. 10.1126/science.1185231 20813952

[6] Bond, R. M. *et al.* A 61-million-person experiment in social influence and political mobilization. *Nature* **489**, 295–298, 10.1038/nature11421 (2012). Publisher: Nature Publishing Group. 10.1038/nature11421 PMC3834737 22972300

[7] Granovetter, M. S. The Strength of Weak Ties. *American Journal of Sociology* **78**, 1360–1380, 10.1086/225469 (1973). Publisher: The University of Chicago Press.

[8] Magnani, M. & Rossi, L. The ML-model for multi-layer social networks. In *Proceedings - 2011 International Conference on Advances in Social Networks Analysis and Mining, ASONAM 2011*, 10.1109/ASONAM.2011.114 (2011).

[9] De Domenico, M. et al. Mathematical Formulation of Multilayer Networks. *Physical Review X* **3**, 041022. 10.1103/PhysRevX.3.041022 (2013).

[10] Kivela, M. et al. Multilayer networks. *Journal of Complex Networks* **2**, 203–271. 10.1093/comnet/cnu016 (2014).

[11] Dickison, M. E., Magnani, M. & Rossi, L. *Multilayer Social Networks* (Cambridge University Press, 2016).

[12] Bianconi, G. *Multilayer Networks: Structure and Function* (Oxford University Press, 2022).

[13] van der Laan, J., de Jonge, E., Das, M., Te Riele, S. & Emery, T. A Whole Population Network and Its Application for the Social Sciences. *European Sociological Review* **39**, 145–160. 10.1093/esr/jcac026 (2023).

[14] Bokányi, E., Heemskerk, E. M. & Takes, F. W. The anatomy of a population-scale social network. *Scientific Reports* **13**, 9209, 10.1038/s41598-023-36324-9 (2023). Publisher: Nature Publishing Group. 10.1038/s41598-023-36324-9 PMC10244344 37280385

[15] Cremers, J. *et al.* Unveiling the social fabric through a temporal, nation-scale social network and its characteristics. *Scientific Reports* **15**, 18383, 10.1038/s41598-025-98072-2 (2025). Publisher: Nature Publishing Group. 10.1038/s41598-025-98072-2 PMC12106742 40419631

[16] Kazmina, Y., Heemskerk, E. M., Bokányi, E. & Takes, F. W. Socio-economic segregation in a population-scale social network. *Social Networks* **78**, 279–291. 10.1016/j.socnet.2024.02.005 (2024).

[17] Hedde-von Westernhagen, C., Bagheri, A. & Garcia-Bernardo, J. Predicting COVID-19 infections using multi-layer centrality measures in population-scale networks. *Applied Network Science* **9**, 1–27, 10.1007/s41109-024-00632-4 (2024). Number: 1 Publisher: SpringerOpen.

[18] Menyhért, M. *et al.* Connectivity and community structure of online and register-based social networks. *EPJ Data Science* **14**, 1–19, 10.1140/epjds/s13688-025-00522-4 (2025). Number: 1 Publisher: SpringerOpen.

[19] Mislove, A., Marcon, M., Gummadi, K. P., Druschel, P. & Bhattacharjee, B. Measurement and analysis of online social networks. In *Proceedings of the 7th ACM SIGCOMM conference on Internet measurement*, IMC ’07, 29–42, 10.1145/1298306.1298311 (Association for Computing Machinery, New York, NY, USA, 2007).

[20] Sala, A., Gaito, S., Rossi, G. P., Zheng, H. & Zhao, B. Y. Revisiting Degree Distribution Models for Social Graph Analysis, 10.48550/arXiv.1108.0027 (2011). ArXiv:1108.0027 [cs].

[21] Park, P. S., Blumenstock, J. E. & Macy, M. W. The strength of long-range ties in population-scale social networks. *Science* **362**, 1410–1413, 10.1126/science.aau9735 (2018). Publisher: American Association for the Advancement of Science. 10.1126/science.aau9735 30573627

[22] Sridharan, A., Gao, Y., Wu, K. & Nastos, J. Statistical behavior of embeddedness and communities of overlapping cliques in online social networks. In *2011 Proceedings IEEE INFOCOM*, 546–550, 10.1109/INFCOM.2011.5935223 (2011). ISSN: 0743-166X.

[23] Borgatti, S. P., Mehra, A., Brass, D. J. & Labianca, G. Network Analysis in the Social Sciences. *Science* **323**, 892–895. 10.1126/science.1165821 (2009). 19213908 10.1126/science.1165821

[24] Tóth, G. *et al.* Inequality is rising where social network segregation interacts with urban topology. *Nature Communications* **12**, 1143, 10.1038/s41467-021-21465-0 (2021). Number: 1 Publisher: Nature Publishing Group. 10.1038/s41467-021-21465-0 PMC7892860 33602929

[25] Panayiotou, G., Magnani, M. & Pinaud, B. Current challenges in multilayer network engineering. *Applied Network Science* **9**, 1–23, 10.1007/s41109-024-00686-4 (2024). Publisher: SpringerOpen.

[26] Panayiotou, G. & Magnani, M. Fair-mod: Fair Modular Community Detection. In Cherifi, H., Donduran, M., Rocha, L. M., Cherifi, C. & Varol, O. (eds.) *Complex Networks & Their Applications XIII*, 91–102, 10.1007/978-3-031-82435-7_8 (Springer Nature Switzerland, Cham, 2025).

[27] Braha, D., Stacey, B. & Bar-Yam, Y. Corporate competition: A self-organized network. *Social Networks* **33**, 219–230. 10.1016/j.socnet.2011.05.004 (2011).

[28] Wohlert, I. K. *Creation and Analysis of a Population-Scale Social Network Based on Swedish Registry Data*. IT ; mDA 24016 (Uppsala University, 2024).

[29] Angriman, E., van der Grinten, A., Hamann, M., Meyerhenke, H. & Penschuck, M. Algorithms for Large-Scale Network Analysis and the NetworKit Toolkit. In Bast, H., Korzen, C., Meyer, U. & Penschuck, M. (eds.) *Algorithms for Big Data: DFG Priority Program 1736*, 3–20, 10.1007/978-3-031-21534-6_1 (Springer Nature Switzerland, Cham, 2022).

[30] Takes, F. W. & Kosters, W. A. Determining the diameter of small world networks. In *Proceedings of the 20th ACM international conference on Information and knowledge management*, CIKM ’11, 1191–1196, 10.1145/2063576.2063748 (Association for Computing Machinery, New York, NY, USA, 2011).

